# WES-Based Screening of a Swedish Patient Series with Parkinson’s Disease

**DOI:** 10.3390/genes16121482

**Published:** 2025-12-10

**Authors:** Efthymia Kafantari, Kajsa Atterling Brolin, Joel Wallenius, Maria Swanberg, Andreas Puschmann

**Affiliations:** 1 Division of Neurology, Department of Clinical Sciences Lund, Lund University, 221 00 Lund, Sweden; 2 Department of Neurology, Skåne University Hospital, 221 00 Lund, Sweden; 3 Translational Neurogenetics Unit, Department of Experimental Medical Science, Lund University, 221 00 Lund, Sweden; 4 Centre for Preventive Neurology, Wolfson Institute of Population Health, Queen Mary University of London, London E1 4NS, UK; 5 Science for Life Laboratory, Department of Clinical Sciences Lund, Lund University, 221 00 Lund, Sweden

**Keywords:** Parkinson’s disease, whole-exome sequencing, genetic investigation

## Abstract

**Background/Objective:** Genetic factors contribute significantly to Parkinson’s disease (PD), especially in cases with early onset or positive family history. However, previous investigations of the genetic landscape in PD populations were mainly based on targeted genotyping. The aim of this study was to investigate the prevalence of pathogenic variants in known PD-associated genes in a series of Swedish PD patients. **Methods:** We performed whole-exome sequencing on 285 PD probands from southern Sweden. Our series was enriched for patients with early disease onset or positive family history. We focused on 44 genes previously linked to PD. **Results:** We identified a *CHCHD2* p.(Phe84LeufsTer6) frameshift variant in two unrelated patients and report the first PD case of Swedish ancestry carrying the *VPS35* p.(Asp620Asn) variant. Additionally, in one patient each, we found an *SNCA* duplication, an *SNCA* p.(Ala53Thr) variant, and a *LRRK2* p.(Gly2019Ser) variant. Thus, only 2.1% (*n* = 6) of patients in this series had Mendelian monogenic PD forms. In addition, forty-three patients carried variants in *GBA1*, including T369M, which may lack disease-association in our population (*n* = 12); E326K (*n* = 22), which is classified as a PD risk variant; as well as N370S (*n* = 3), R329H (*n* = 3), S107L (*n* = 1), and L444P (*n* = 1), with one patient harboring both T369M and E326K. Pathogenic variants in *ARSA*, *ATP7B*, and *PRKN* genes were also detected in heterozygote form, but their role in PD remains uncertain. **Conclusions:** Monogenic forms of PD are rare in southern Sweden, even among the familial and early-onset PD patients that were overrepresented in our study. Our findings highlight the genetic diversity in Swedish PD patients and identify key variants for further functional and clinical studies.

## 1. Introduction

The etiology of Parkinson’s disease (PD) is considered to be multifactorial, including complex interactions of genetic and environmental factors. In most cases, PD occurs sporadically in patients who are not aware of any family members with the disease. However, PD can be familial, as 10–15% of patients report a positive family history with at least one affected first- or second-degree relative. Mendelian forms of PD have been reported in about 5–10% of cases internationally and are caused by rare variants in a small number of genes [1]. In these Mendelian forms, a variant in one gene is sufficient to cause PD (monogenic PD), and these variants are of high impact. Some of the genes are associated with autosomal dominant patterns (e.g., *SNCA*, *LRRK2*, and *VPS35*) and others with recessive patterns of inheritance (e.g., *PRKN*, *PINK1*, and *PARK7*). Alterations in *GBA1* do not cause Mendelian forms of PD but are considered the most important genetic risk factor for developing PD, as between approximately 5% and 15% of people with PD carry *GBA1* variants [2]. The frequency of *GBA1* variants differs in different populations; for example, in the European Ashkenazi Jewish population, the frequency is 10–31% [2]. The penetrance of *GBA1* variants for PD is low and depends on the variant and other genetic variation [3].

Recent advances in genetic testing methods have greatly facilitated a broader investigation of the genetic background of PD. In the case of familial PD, next-generation sequencing (NGS) methods can be applied to screen patients for monogenic forms. Most of the known disease-causing variants are located in exons [4] and can thus be captured by whole-exome sequencing (WES). Here, we aimed to investigate the occurrence of monogenic PD in a patient series from a limited geographical area in southern Sweden including PD patients with a young age at onset and/or positive family history, using WES.

## 2. Methods

### 2.1. Patient Selection

We studied 285 clinically well-characterized PD patients from southern Sweden. All patients provided informed written consent in research studies on genetics of PD. Information about disease and family history was obtained. Patients had been included in the PARkinsonLUnd (PARLU) study [5] or the MultiPark biobank sample collection (MPBC) [6], as previously described. For the PARLU study, a diagnosis of PD was verified through clinical examinations by the authors and longitudinal follow-up within the research study [5]. For MPBC, diagnosis was verified through national Parkinson registry data [7], which is based on information from the treating neurologists. From among the 1150 patients included in these studies combined, we report findings from WES studies of 285 patients here.

Patients from the PARLU study were selected for WES analysis based on the time when they were included in the study. Patients from the MPBC study were selected based on their age of onset or because they reported positive family history with at least one first- or second-degree relative with PD; we preferentially sequenced individuals with a larger number of affected relatives. The total number of sequenced individuals was limited for financial reasons. We did not sequence more than one patient per family.

### 2.2. Genetic and Bioinformatic Analyses

Blood was drawn and DNA was extracted following standard procedures. WES was performed within the MultiPark NGS database (https://www.multipark.lu.se/infrastructures/ngs-database-infrastructure, accessed on 22 September 2025) infrastructure on an Illumina platform at the Center for Translational Genomics, Lund University. A diagram of the bioinformatic workflow is provided as Supplemental Figure S1. WES data were mapped to the GRCh38/hg38 reference genome and used for the analysis of single nucleotide variants (SNVs), small insertions and deletions (indels), and copy number variants (CNVs). By using the Genome Analysis Toolkit (GATK v4.3.0.0) [8], variant calling format (VCF) files were produced. The obtained VCF files were evaluated with GATK and bcftools v.1.9 [9] and annotated with Variant Effect Predictor v109 (VEP) by Ensembl [10]. A filtering strategy was followed where variants with a minor allele frequency (MAF) < 0.02 in gnomAD, non-Finnish European exomes and genomes [11], and a genotype quality of more than 20 were selected. The frequency of variants was also checked in the 1000 Genomes Project EUR ancestry group [12]. Variants in 44 genes associated with PD (Human Phenotype Ontology [https://hpo.jax.org/] term: HP:0002548 and newly discovered genes reported in the literature by July 2025, Supplemental Table S1) were assessed further. Missense variants were annotated with the dbNSFP v4.4a [13,14] plugin of VEP and prediction of variants’ effect on splicing was achieved with spliceAI v1.3 [15]. Variants were prioritized based on their CADD-phred score v1.7 [16]. Intronic and synonymous variants were assessed only if the CADD-phred score was above 20; a variant with that CADD-phred score is among the top 1% most deleterious substitutions in the human genome. The variants were searched for in the ClinVar database [17] and classified based on the criteria that were proposed by the American Collage of Medical Genetics and Genomics (ACMG) [18] by using VarSome [19] as well as Franklin by Genoox (https://franklin.genoox.com/clinical-db/home, accessed on 29 September 2025). CNVs were detected with the R package ExomeDepth v1.1.12 [20]. When we identified heterozygous variants in genes for recessive PD, we screened SNV and CNV data for additional variants in the same gene. If no additional SNVs or CNVs were detected in the same gene, we excluded heterozygous genotypes in recessive PD genes from further analysis.

SNVs and small indels in *GBA1* were manually curated with Integrative Genomics Viewer (IGV) v2.16.2 [21] to make sure the calls were true positives, rather than artifacts due to mis-mapped reads from the highly homologous pseudogene *GBAP1*. This was performed using the BLAT [22] tool available in IGV.

For the two patients with the *CHCHD2* frameshift variant, identity-by-descent (IBD) analysis was performed to investigate potential relatedness. VCF files spanning all autosomal chromosomes were phased and imputed with the Michigan Imputation Server 2 [23], using the HRC r.1.1 2016 reference panel [24]. After phasing, hap-ibd v1.0 was used for the IBD analysis [25]. The results were curated with *in-house* scripts.

For confirmation and determination of the breakpoints of the *SNCA* duplications, SNV array analysis was performed at Centogene, Rostock, Germany, using the Infinium™ Global Diversity Array with Cytogenetics kit (Illumina Inc., San Diego, CA, USA). This kit includes approximately 1.8 million SNV markers spread across the entire genome and covers 99.9% of exons.

### 2.3. PD Patients in the MDCS Cohort

To evaluate monogenic variant findings, we used clinical and genetic datasets from the Malmö Diet and Cancer Study (MDCS; https://www.malmo-kohorter.lu.se/malmo-diet-cancer-md, accessed on 22 September 2025), a population-based cross-sectional cohort designed to investigate potential links between dietary/lifestyle factors and cancer incidence. Conducted between 1991 and 1996, the MDCS enrolled 18,275 women (birth years 1923–1950) and 12,108 men (birth years 1923–1945), all residents of Malmö, Sweden. WES had previously been performed for MDCS participants during 2017–2018 [26]. In total, 29,387 individuals had complete genetic and clinical data and were evaluated here. Among them, 695 were affected with PD. Variants were evaluated by the chi-squared test and one-sided Fisher’s exact test, as well as odds ratio calculations on allele frequencies. Because of low numbers of occurrences, the Yates correction of the chi-squared *p*-value was also applied.

## 3. Results

A total of 285 PD patients were included in the study, of which the average age of onset was 56.1 (±16.1; range 20–82) years, 175 of 285 (61%) were females, and 173 (60.8%) had a positive family history of PD. Ninety-one patients had early-onset PD (onset at or before 50 years of age). Descriptive information for the patient series is provided in Table 1.

After WES and initial bioinformatic analyses, the quality of the VCF files was evaluated by calculating specific metrics for which a value out of given ranges could indicate biases and/or artifacts during the analysis. The transitions/transversions ratio was ~3.0, as expected for WES data, and the number of detected variants averaged approximately 3000 indels and 35,000 SNVs for each individual sample.

### 3.1. Known Pathogenic Variants in Established PD Genes

Previously known pathogenic variants were identified in 4 of 285 probands (Table 2). *LRRK2* p.(Gly2019Ser) was found in one proband, and family investigation showed that the proband’s affected mother also carried the same variant. The finding of this patient was reported in a previous study [6].

**Figure 1:**
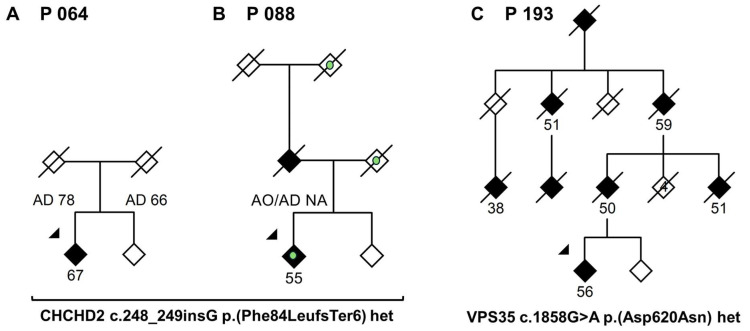
Pedigrees of the families with the *CHCHD2* p.(Phe84LeufsTer6) (**A**,**B**) and the *VPS35* p.(Asp620Asn) (**C**) variants. Black diamond: affected with PD; white diamond: unaffected; green circle: dementia; arrowhead: proband. The age at disease onset, if known, is shown for the affected individuals. AO = age at onset, AD = age at death, NA = not available.

In one familial PD patient, we identified the *VPS35* p.(Asp620Asn) variant that is confirmed to cause autosomal dominant PD (Table 2, Figure 1). This patient had bilaterally markedly reduced dopamine reuptake in 123I-ioflupane SPECT at age 51 (Supplemental Figure S2) and normal brain MRI—without signs of white matter disease or cerebellar or cerebral atrophy—at age 56. She underwent a successful DBS operation and at age 73 had no overt cognitive dysfunction.

One patient found to carry *SNCA* p.(Ala53Thr) had also already been reported previously in independent work [27]. *SNCA* duplication was detected in one patient; information from the patient and family revealed that this individual belonged to the previously published family from the Lister peninsula in southern Sweden [28]. The exact size of the duplicated segment was determined with a microarray genotyping assay as arr[GRCh37]4q22.1(90320154_91139340)x3 and was very similar to the duplicated genomic segment in another affected family member of the Lister family who had been seen in the authors’ clinic (Supplemental Figure S3).

### 3.2. CHCHD2 p.(Phe84LeufsTer6)

A frameshift variant, p.(Phe84LeufsTer6), was found in *CHCHD2* in two probands with PD (Table 2, Figure 1). The CADD-phred score for this frameshift variant was 31, and the allele frequency in the gnomAD database [11] (exomes and genomes) was 0.0001239% (2 of 1,613,734 alleles), where these two alternate alleles were found in the non-Finnish European genetic ancestry group. This variant had not been previously associated with PD. We searched the large MDCS cohort with WES data on 29,387 residents of the city of Malmö, of whom 695 developed PD, for this variant. In MDCS, the variant was found in 2 (of 25,684) controls but 0 (of 695) PD patients. The MDCS study protocol does not allow the retrieval of personal information on participants, which is why we have been unable to examine these individuals for possible mild clinical signs. Our statistical analysis confirmed that this frameshift variant is clearly associated with PD status; carrying *CHCHD2* p.(Phe84LeufsTer6) inferred 26.2 times higher odds of having PD, and this result is statistically significant (Table 3).

Identity-by-descent (IBD) analysis detected a shared genomic segment of 4.7 Mbp (3.452 centiMorgans) enveloping the entire *CHCHD2* gene that was the longest shared segment between the two investigated patients.

The p.(Phe84LeufsTer6) carrier P 064 was a man who developed resting tremor in his right hand at age 67. He reported no family history of PD, but his mother had died aged 66. He reported nocturnal leg cramps and at a research examination at age 73 years, he had a hoarse voice reminiscent of spasmodic dysphonia. He had rigidity, bradykinesia, stooped posture, and Parkinsonian gait disturbance, and his postural reflexes were reduced. Parkinsonism was responsive to levodopa but the patient had symptomatic orthostatic hypotension when treated at low doses (150 mg/d). He had 17 points in UPDRS part III and had reached Hoehn and Yahr stage III. Non-motor symptoms included constipation, nycturia, muscular pain, erectile dysfunction, symptoms of REM sleep behavior, restless legs, intensified dreams, and depressive symptoms. There was no overt cognitive dysfunction. Brain CT scans of this patient at age 82 showed minimal cerebral atrophy that was considered mostly age-dependent, although influence of the neurodegenerative process of their PD could not be excluded.

The p.(Phe84LeufsTer6) carrier P 088 was a woman who developed resting tremor at approximately 55 years of age. She reported that her father had had PD with tremor, stiffness, and slowness of bodily movements, gait disturbance, and in the terminal disease stage had become unable to feed himself. Her mother and her father’s mother both had dementia. On examination at age 70, she had marked positional tremor, and bilateral bradykinesia that was more pronounced on her right side, reduced postural reflexes but no marked rigidity. Her gait was slightly slow and broad-based, ascribed to a marked fear of falling. Handwriting was slow and micrographic. The patient reported restless leg symptoms and morning leg dystonia. Her Hoehn and Yahr stage was Ib. She developed cognitive dysfunction and repeated Mini-Mental State examinations showed 19 of 30 points at age 78 and 16 points at age 80. At age 76 she moved to a dedicated nursing home for the care of people with cognitive dysfunction. Dementia was diagnosed. On her most recent examination at age 80 years, she had 17 points in UPDRS-III and Hoehn and Yahr stage IV. Several brain CT scans performed between ages 73 and 82 showed increasing cerebral atrophy especially in the topmost scan slices and moderate periventricular white matter hyperdensity.

### 3.3. Overall Diagnostic Yield for Monogenic PD

Combining our findings of four known pathogenic variants with two patients with *CHCHD2* p.(Phe84LeufsTer6), a positive finding was obtained for 2 (8%) of 25 PD patients with young onset and positive family history, 2 (2.2%) of 91 early-onset PD patients, 5 (2.9%) of 173 patients with positive family history, and 1 (0.9%) of 112 patients with negative family history. In the entire series, 6 (2.1%) of 285 patients had a monogenic form of PD.

### 3.4. GBA1 Risk Variants

**Figure 2:**
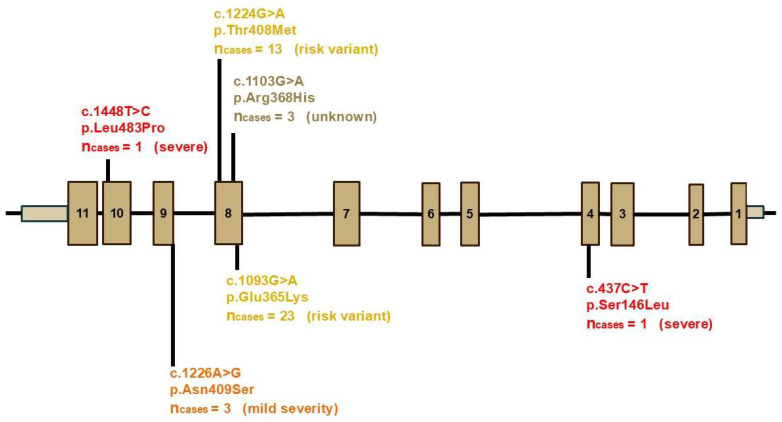
*GBA1* variants identified in 285 probands with PD from Sweden. In parenthesis, the severity of the variants is shown, based on the classification proposed by Parlar et al. [29] (https://pdgenetics.shinyapps.io/gba1browser/, accessed on 29 September 2025). Image adapted from Gabbert et al. [32] (http://creativecommons.org/licenses/by/4.0/, accessed on 29 September 2025) with modifications. Numbered brown boxes represent *GBA1* exons and the black lines represent the introns. Risk variants are highlighted in yellow, while variants of unknown, mild and high severity are shown in dark yellow, orange and red, respectively.

We identified variants in *GBA1* that had been reported [29] to be associated with PD risk in 43 (15.1%) of 285 probands (Figure 2) and list them here with their classification as “risk variant”, “mild”, “unknown severity”, and “high severity” following the GBA1 browser classification [29]. The risk variants p.(Thr408Met), previous designation: T369M, and p.(Glu365Lys), E326K were found in 12 and 22 probands, respectively; one individual carried both these risk variants. Analysis of the 131 samples from the MPBC for the p.(Thr408Met), T369M variant have previously been reported [30]. As two studies recently revealed a lack of association of p.(Thr408Met) with PD in the Swedish population, we recalculated the frequency of *GBA1* variants in our patients without this variant; this revealed that 10.9% of patients carried any of the other *GBA1* variants. The variant p.(Asn409Ser), N370S of mild severity was detected in three probands and the p.(Arg368His), R329H of unknown severity in three others. Two variants with high severity, the p.(Ser146Leu), S107L, and the p.(Leu483Pro), L444P, were also identified in one proband each. Published odds ratios (ORs) of the variant p.(Leu483Pro), L444P fall within the range of 6.40 to 30.4 [29], showing that the variant is strongly associated with an increased risk of PD. The association of the heterozygous p.(Ser146Leu), S107L variant with early-onset PD was described for the first time in 2019 when it was found in two Swedish half-siblings [31]. All variants were confirmed to stem from *GBA1* and had not been mis-mapped to the pseudogene *GBAP1*.

### 3.5. Heterozygous ATP7B Variants

We identified the *ATP7B* variants p.(His1069Gln) and p.(Gln289Ter) in one proband each and p.(Thr991Met) in two probands (Supplemental Table S2). In bi-allelic genotypes, all these variants cause Wilson’s disease [33,34] with recessive inheritance, but all carriers in the present study were heterozygous for these variants and our analyses revealed no additional variant in *ATP7B* in these individuals.

### 3.6. Variants in ARSA

Four heterozygous *ARSA* variants were found in our patient series, with classifications ranging from variants of uncertain significance (VUSs) to pathogenic according to the ACMG criteria [18], based on the ClinVar database [17] and the classification tools VarSome [19] and Franklin by Genoox (https://franklin.genoox.com/clinical-db/home). The *ARSA* p.(Pro351Ala) variant (*n* = 1 proband) is extremely rare in the gnomAD database (0.000063%) and currently classified as a VUS. The *ARSA* p.(Arg301Gln) variant (*n* = 1) is also considered a VUS in ClinVar but is classified as likely pathogenic by Franklin by Genoox. Another missense variant, *ARSA* p.(Gly129Ala) (*n* = 1), is classified as pathogenic by VarSome and is absent from the gnomAD database, and the splicing variant *ARSA* c.465 + 1G>A (*n* = 2) is classified as pathogenic in ClinVar. Further details on these variants are provided in Supplemental Table S2. We also searched the MDCS cohort for the *ARSA* variants identified in this study. *ARSA* p.(Gly129Ala) had a strong association with PD while the remaining three variants had no significant association and a relatively high frequency in the control group (Table 3).

### 3.7. Additional Variants

We identified several variants of uncertain significance and unknown disease-variant association (Supplemental Table S2). These included monoallelic variation in genes associated with autosomal recessive PD, such as *VPS13C* and *PRKN*, and variants in genes where only one (other) variant has been associated with PD until now, such as *RAB32* p.(Ser71Arg).

In *PRKN*, we identified four different heterozygous variants in four probands. They included p.(Arg275Trp), classified as pathogenic based on the ClinVar database, p.(Arg275Gln) and p.(Arg256Cys) of uncertain significance, and p.(Gly429Glu), which has not been reported in ClinVar. They were found in one case each (Supplemental Table S2).

## 4. Discussion

We performed a comprehensive WES-based analysis of both single nucleotide variants (SNVs) and copy number variants (CNVs) in 44 PD-related genes in 285 PD probands from southern Sweden. Our series was markedly enriched for patients who reported a positive PD family history (60.8%) and patients with a younger age at disease onset (31.9%)—groups where the genetic component to the disease etiology cause is likely increased. Despite this, only 6 out of 285 (2.1%) probands with monogenic PD carrying disease-causing variants were identified. We assume that the true prevalence of monogenic PD that can be detected by WES among unselected Swedish PD patients is lower. We have previously reported a low frequency (0.59%) of known monogenic variants in a large Swedish series of PD patients (*n* = 2206) screened for variants and/or duplication in *SNCA* and *LRRK2* [35]; a subgroup of the 154 probands from the present study were included in that study [35]. In contrast, a recent large screening study on 8301 North American patients reported 272 (3.3%) individuals with monogenic PD genotypes (including only disease-causing variants), which was largely driven by 196 (2.4%) carriers of *LRRK2* variants, mostly p.(Gly2019Ser) [36]. Another genetic analysis conducted in the United Kingdom found known pathogenic variants in 69 of 718 screened PD families and early-onset patients (9.6%) [37]. These frequences are higher than the frequences of monogenic PD causes that have been identified in Sweden.

Our study identified variants in *SNCA*, *LRRK2*, and *VPS35* that are well-established causes for PD.

*SNCA* p.(Ala53Thr) was the first genetic variant ever described to cause PD [38]. Previously, our group described a Swedish PD family carrying a de novo *SNCA* p.(Ala53Thr) variant [27]; one of these carriers was included in the present study.

Duplications and triplications in *SNCA* have been described in individuals with PD from different populations [39,40]. Over 47 families and 18 sporadic cases have been documented with variable breakpoints, suggesting multiple independent founder events [41,42]. In 2007, a family of Swedish origin (the large “Lister family”) was found to carry an *SNCA* duplication [28]. In our study, we identified one PD patient with a duplication spanning the *SNCA*, *SNCA-AS1*, the neighboring *MMRN1,* and ~91 kb of *CCSER1*. Comparing high-resolution graphs of the SNV assay of this patient with that of a confirmed carrier in the Lister family revealed that they belong to the same family (Supplemental Figure S3).

In our study, one proband (0.35%) carried the *LRRK2* p.(Gly2019Ser) variant, the most common PD-associated *LRRK2* variant internationally, exceeding a frequency of 1.0% among PD patients in 26 out of 51 countries [43]. This variant exhibits reduced penetrance; therefore, not all carriers develop PD. It is much less common in Sweden and Northern European populations compared to other studied regions; thus far in Sweden, only a small number of PD patients with the variant have been reported [35,44].

The *VPS35* p.(Asp620Asn) variant is the only confirmed pathogenic variant in the *VPS35* gene known to date [45,46,47,48]. Our study is, to our knowledge, the first to detect *VPS35* p.(Asp620Asn) in a PD patient with Swedish ancestry. The patient’s and the affected relatives’ clinical phenotype was similar to previous descriptions of *VPS35* p.(Asp620Asn), with a relatively early age at onset of 39–51 years (Figure 1C).

We found the *CHCHD2* frameshift variant p.(Phe84LeufsTer6) in two probands who both originated from a small geographic area within our center’s catchment area in southern Sweden. However, the two individuals did not share genomic segments longer than 3.4 cM. This suggests that the individuals displayed higher genetic similarity than the general population-level ancestry but that they likely were not closely related, because closely related individuals usually share much longer DNA segments [49].

CHCHD2 is a small mitochondrial protein involved in mitochondrial homeostasis [50]. In 2015, two missense *CHCHD2* variants, p.(Thr61Ile) and p.(Arg145Gln), and the splice site *CHCHD2* variant c.300 + 5G>A were identified in Japanese families with late-onset autosomal dominant PD [51] and later these variants were found in PD patients of Chinese origin [50]. Since then, more and more *CHCHD2* variants have been found in Asian PD cases. Fifteen rare exonic *CHCHD2* variants have been observed in Caucasian patients from which only two, p.(Pro2Leu) and p.(Ala32Thr), have been found in Asian populations [50]. Notably, p.(Ala71Pro) was found in a homozygous state in an early-onset PD patient [52].

One nonsense variant, *CHCHD2* p.(Gln126Ter), was identified in a German PD patient [53], and one, *CHCHD2* p.(Tyr99Ter), in an Eastern Chinese patient [54]. In two Chinese PD patients of early onset, the *CHCHD2* p.(Pro53AlafsTer38) frameshift variant in exon 2 was found [55]. This frameshift variant was also present in three Chinese PD patients of late onset from an independent cohort [55]. Experimental data showed that truncating *CHCHD2* frameshift and nonsense variants lead to loss of function, likely because of nonsense-mediated RNA decay, and are linked to mitochondrial dysfunction [55]. The *CHCHD2* p.(Phe84LeufsTer6) variants in the two probands in our series likely have the same loss of function effect and thus are classified as likely pathogenic according to ACMG criteria [16].

The phenotype of P 064 was that of typical late-onset PD with resting tremor at onset but subsequent development of postural instability and gait disturbance, but no overt cognitive dysfunction. P 088 had marked action tremor for many years before Parkinsonian signs developed. Similarly, in one of the original Japanese families with *CHCHD2* p.(Thr61Ile), one variant carrier had a diagnosis of essential tremor and not of PD [51]. Additionally, patient P 088 developed dementia. Marked cognitive decline or dementia are not known to be early or prominent features in patients with *CHCHD2*-related PD [51,56], but milder cognitive dysfunction has been described in some of the patients [55], and there is still a relative paucity of descriptions of patients in terminal disease stages. Possibly, P 088 had inherited a susceptibility to dementia from her mother and PD from her father.

In a large international cohort, 10.4% of individuals carried a *GBA1*-variant associated with PD [57]. The frequencies of p.(Thr408Met), T369M, and p.(Glu365Lys), E326K in our series were 4.5% and 8%, respectively. In reported studies on White/Caucasian populations combined, the frequencies of p.(Thr408Met), T369M, and p.(Glu365Lys), E326K were 2.03% and 4.07%, respectively [32]. Two previous studies have shown that p.(Thr408Met), T369M is not associated with PD in the Swedish population [30,58], but this variant is listed in the GBA1 browser [29] which we used in our study to decide which variants to count. Including p.(Thr408Met), T369M, 43 of 285 patients (15.1%) carried a *GBA1* variant; excluding p.(Thr408Met), T369M, 10.9% of patients carried one of the other *GBA1* variants. The majority of the remaining *GBA1* variant carriers had p.(Glu365Lys), E326K, which is only classified as a risk variant [29].

The *GBA1* p.(Asn409Ser), N370S variant was identified in 1% of our probands, which is relatively higher than in a previous Swedish study (0.62%) [59]. The frequency of this variant was 1.59% in reported studies on White/Caucasian populations combined [32].

The frequency of *GBA1* p.(Leu483Pro), L444P, found in 0.35% of our patient series, deviated significantly from the 2.15% found in another Swedish study [59]. We assume that this reflects that our patients are more remote from the known Northern Swedish/Norrbottnian founder [60]. In Europeans this variant’s frequency was 1.53% in reported studies on White/Caucasian populations combined [32]. The p.(Arg368His), R329H variant of unknown significance found in three unrelated probands (1%) has previously only rarely been reported [57,61].

The *GBA1* p.(Ser146Leu), S107L variant was identified in one (0.35%) proband with an age at onset at 66 years, who had reported one affected parent and one additional affected family member. The same variant had been described in a small number of Gaucher patients in compound heterozygous state with other *GBA1* severe variants and was detected in two Swedish heterozygous PD half-siblings with a severe PD phenotype [31].

Lysosomal storage disorders are a group of human diseases arising from recessive mutations in genes essential for lysosomal function. More than two-thirds of these disorders present with neurological complications, such as Parkinsonism [62]. Recent research has revealed a close link between lysosomal storage disorders and several common neurodegenerative diseases. The most well-established example is the association between mutations in *GBA1* and PD. *ARSA* is one of these lysosomal genes. Both *GBA1* and *ARSA* encode important enzymes in the lysosomal ceramide pathway. *ARSA* encodes a lysosomal enzyme that breaks down sulfatide into galactosylceramide [62]. Biallelic *ARSA* pathogenic variants cause metachromatic leukodystrophy, while heterozygous *ARSA* variants were first linked to PD in 2019 [62]. Rare *ARSA* loss-of-function variants were associated with PD in a recent study [63] and a multiethnic burden analysis also suggested a link between functional *ARSA* variants and PD, though associations lost significance after correction for multiple comparisons [64]. The splicing variant c.465 + 1 G>A, found in our patient series, may be linked to PD, but further genetic and functional studies are needed to confirm its role [64].

We thus assessed the possible association of heterozygous *ARSA* variants with PD in a large population-based dataset. Based on our findings, only the *ARSA* p.(Gly129Ala) had a strong association with PD. However, this finding should be interpreted with caution as the gene’s association with PD remains to be fully confirmed. Odds ratios calculated for the variants (Table 3) may be mildly overestimated because we, with one exception, combined observations from our patient series with the MDCS database; however, due to the paucity of observations, we saw no other way to determine odds ratios.

Our analysis also identified carriers of heterozygous variants in recessive genes, including pathogenic *ATP7B* variants, which are associated with Wilson’s disease in a homozygous or compound heterozygous state. It has been suggested that heterozygosity in *ATP7B* may phenotypically manifest as PD [65] but this requires further exploration. Similarly, heterozygous variants in the *PRKN* gene are considered to be potential genetic risk factors for PD [66] and were found to be more frequent in PD cases than in controls [67], but there remain uncertainties about heterozygous *PRKN* variants with PD. Moreover, although we searched for the presence of a second variant in the same gene in our analyses, we cannot entirely rule out other types of variants not detected by WES technology.

## 5. Conclusions

In our study, the largest WES-based investigation of PD patients conducted in Sweden, a clear disease-causing variant was identified in only 2.1% of cases (six individuals) in *SNCA*, *LRRK2*, *VPS35,* and *CHCHD2* genes, although our series was enriched for patients with early onset or positive family history in whom a monogenic disease cause a priori is more likely. A positive finding was obtained for 2 (8%) of 25 PD patients with young onset and positive family history, 2 (2.2%) of 91 early-onset PD patients, 5 (2.9%) of 173 patients with positive family history, and 1 (0.9%) of 112 patients with negative family history. This low yield may be attributed to the use of WES, which is limited to coding regions and does not capture non-coding, deep intronic, or regulatory variants. It also reflects the complex etiology of PD, which is not always driven by monogenic variants, even in individuals with early disease onset and/or a positive family history. Rather, di-, oligo-, or polygenic factors, alone or in combination with environmental risk factors, may explain the occurrence of PD and its clustering in several individuals of some families in the population. Our findings confirm the pathogenicity of truncating variants in *CHCHD2* and thus more clearly associate this gene with autosomal dominant PD.

## Figures and Tables

**Table 1:** Description of the study population.

**Total Study Population (PARLU and MPBC Studies Combined):**	
Number of probands	285
Females/males	61%:39%
Age at symptom onset (mean ± SD; range)	56.1 (±16.1; 20–82) years
Age at study inclusion/last clinical follow-up (mean ± SD)	65.5 (±11.4) years
Positive family history (any family member had PD)	173 (60.8%)
Early onset (at or before age 50)	91 (31.9%)
Mean (median; range) age of onset among the 91 patients with early onset	43.2 (45; 20–50) years
**Composition of PARLU and MPBC studies:**	
**PARLU study**	**Number of probands** **(with positive family history)**
Total	154 (95)
≤30 years of age at symptom onset	1 (1)
>30 and ≤40 years of age at symptom onset	5 (0)
>40 and ≤50 years of age at symptom onset	20 (10)
>50 years of age at symptom onset	116 (78)
unknown age at symptom onset	12 (6)
**MPBC study**	**Number of probands** **(with positive family history)**
Total	131 (78)
≤30 years of age at symptom onset	2 (1)
>30 and ≤40 years of age at symptom onset	18 (1)
>40 and ≤50 years of age at symptom onset	45 (10)
>50 years of age at symptom onset	54 (54)
unknown age at symptom onset	12 (12)

MPBC: MultiPark’s biobank sample collection, nr: number, PARLU: PARkinsonLUnd study.

**Table 2:** List of patients carrying disease or likely disease-causing variants in genes that are associated with PD.

Proband ID (Sex)	Age at Onset (Years)	Positive Family History	Phenomenology	Variant(s) Identified	ClinVar Entries (ID #), CADD-Phred Score, ACMG Classification	Interpretation
P038 (F)	39–41	Yes	Parkinsonism, cognitive decline, language deficits, dysautonomia PMID: 19632874	***SNCA*** c.157G>A p.(Ala53Thr) het	P (14007), 15.7, P (PS4, PP1, PS3, PM1, PP2, PM2, PM5)	Disease-causing, known pathogenic variant.
P221 (F)	43	Yes	Parkinsonism, rigidity, bradykinesia in left side	***LRRK2*** c.6055G>A p.(Gly2019Ser) het	P/LP; risk factor (1940), 31, P (PS4, PP3, PM2)	Disease-causing, known pathogenic variant.
P193 (F)	56	Yes	Parkinsonism. Marked family history for PD and cognitive decline, average AAO in family: 50.8 years	***VPS35*** c.1858G>A p.(Asp620Asn) het	Not reported, 31, LP (PS4, PP1, PM2, PP2)	Disease-causing, known pathogenic variant.
P064 (M)	67	No	Parkinsonism, tremor, dystonic signs (see main text)	***CHCHD2*** c.248_249insG p.(Phe84LeufsTer6) het	Not reported, 32, LP (PVS1, PM2)	Likely disease-causing, novel variant.
P088 (F)	57	Yes	Parkinsonism, tremor, dystonic signs, dementia (see main text)	***CHCHD2*** c.248_249insG p.(Phe84LeufsTer6) het	Not reported, 32, LP (PVS1, PM2)	Likely disease-causing, novel variant.
P182(F)	51	Yes	Cognitive dysfunction, Parkinsonism, dysautonomia. Unilateral upper limb spasticity in advanced disease	***SNCA*** ExomeDepth [GRCh38] (chr4:89724100-89954614)x3 arr [GRCh37] 4q22.1(90320154_91139340)x3	Not reported	Disease-causing, known pathogenic variant

CADD: Combined Annotation Dependent Depletion; F: Female; M: Male; het: heterozygous; ACMG: American Collage of Medical Genetics and Genomics. Nomenclature refers to NM_000345.4 (*SNCA*), NM_198578.4 (*LRRK2*), NM_016139.4 (*CHCHD2*), and NM_018206.6 (*VPS35*).

**Table 3:** Association of variants identified in this study with PD, based on observations in the MultiPark NGS database and the MDCS database combined.

**Variant(s) Identified** **gnomAD Frequency NFE**	**Occurrence in This Study** Number of Carriers from Among 285 Probands with PD *	**Occurrence in MDCS Population Database** Number of Carriers from Among 695 PD Patients and 25,684 Controls *	**Chi-Squared *p*-Value, ** **One-Sided Fisher’s Exact Test *p*-Value, ** **OR (95% CI),** **of All Individuals Combined**
***CHCHD2*** c.248_249insG p.(Phe84LeufsTer6) het 0.000001695	2	0 PD, 2 controls	*p*: 0.00000086 (0.00032 Yates-corrected), *p*: 0.0077, OR: 26.23 (3.69–186)
***ARSA*** c.386G>C p.(Gly129Ala) het	1	0 PD, 0 controls	*p*: 0.00000031 (0.013 Yates-corrected), *p*: 0.037, OR: 78.58 (3.19–1930)
***ARSA*** c.465 + 1 G>A het 0.001030	2	3 PD, 81 controls ^§^	*p*: 0.29 (0.44 Yates-corrected), *p*: 0.21, OR: 1.61 (0.65–4.00)
***ARSA*** c.902G>A p.(Arg301Gln) het 0.00001441	1	0 PD, 13 controls	*p*: 0.49 (1.0 Yates-corrected), *p*: 0.41, OR: 2.01 (0.26–15.41)
***ARSA*** c.1051C>G p.(Pro351Ala) het 0.000	1	0 PD, 6 controls	*p*: 0.13 (0.62 Yates-corrected), *p*: 0.23, OR: 4.36 (0.52–36.30)

The Yates correction of the chi-squared *p*-value was performed because of low numbers of observations. CI: 95% confidence interval; OR: odds ratio; het: heterozygous; NFE: Non-Finnish Europeans. * Detailed contingency table is provided as Supplemental Table S3. ^§^ analysis within the MDCS dataset showed chi-squared *p*: 0.59 (0.84 Yates-corrected), Fisher’s exact *p*: 0.38, OR: 1.37 (0.43–4.34).

## Data Availability

The dataset analyzed during the current study is not publicly available due to patient integrity and local and national Swedish laws. However, the corresponding author or andreas.puschmann@med.lu.se can be contacted with requests for potential collaborative studies. The scripts that were used for the analysis of the 285 PD patients can be found in the public domain on GitHub. (https://github.com/efi-ka/286_parkinson, accessed on 29 September 2025).

## Supplementary Materials

The following supporting information can be downloaded at: https://www.mdpi.com/article/10.3390/genes16121482/s1, Table S1: The 44 Parkinson disease related genes that were searched for variations in this study. Table S2: Variants of unknown significance identified in this study with a CADD_phred score above 20. Table S3: Contingency tables for the association tests reported in Table 3. Figure S1: Workflow diagram of the bioinformatic analyses. Figure S2: 123I-ioflupane SPECT of patient P193 with VPS35 p.(Asp620Asn) at age 51. Figure S3: Graphs of SNV array assay of *SNCA* duplications identified in one member of the Lister family (A) and in the PD patient of this study (B). References [62,68,69,70,71,72,73,74,75,76] are cited in the Supplementary Materials.
