# Supplementary material for: WES-Based Screening of a Swedish Patient Series with Parkinson’s Disease
Source: Genes (Basel). 2025 Dec 10;16(12):1482. doi: 10.3390/genes16121482 (PMC12732595; doi:10.3390/genes16121482)
Supplement: Supplementary file 1 [file genes-16-01482-s001.zip › genes-4023685-supplementary.pdf]

Supplemental data

WES-Based Screening of a Swedish Patient Series with Parkinson's Disease

Efthymia Kafantari, Kajsa Atterling Brolin, Joel Wallenius, Maria Swanberg, Andreas Puschmann

Supplemental Table S1: The 44 Parkinson disease related genes that were searched for variations in this study.

ANKRD11	*EPG5*	*PDE10A*	*SNCA*
ARSA	*FBXO7*	*PINK1*	*SYNJ1*
ATP13A2	*GBA1*	*PLA2G6*	*TAF1*
ATP7B	*GCH1*	*POLG*	*TH*
BORCS5	*GIGYF2*	*PRKN*	*TK2*
CHCHD2	*HTRA2*	*PSAP*	*TMEM230*
DAGLB	*ITSN1*	*PSMF1*	*TWNK*
DCTN1	*LRP10*	*PTPA*	*UCHL1*
DNAJC13	*LRRK2*	*PTRHD1*	*UQCRC1*
DNAJC6	*MAPT*	*RAB32*	*VPS13C*
EIF4G1	*PARK7*	*RAB39B*	*VPS35*

The gene list was compiled by retrieving information from Human Phenotype Ontology (HP:0002548 entry) and from original articles that identified new genes as potential candidates for PD [1-10].

Supplemental Table S2: Variants of unknown significance identified in this study with a CADD_phred score above 20.

Sample ID	Chr	Position	Ref allele	Alt allele	Gene name	Existing variation	Consequence	HGVSc	HGVSp	gnomAD genomes NFE	gnomAD exomes NFE	Exon/ Intron	SIFT	Polyphen	CADD phred score	Zygosity	ClinVar clinical significance	VARSOME (ACMG)	Franklin by Genoox ACMG Classification
**01**	3	184327401	G	A	EIF4G1	rs112176450&CM117009&COSV59992434	Missense variant	ENST00000346169.7:c.3614G>A	ENSP00000316879.5:p.Arg1205His	0.0003675	0.0003825	24/33	Deleterious low confidence (0)	Benign (0.174)	27.1	het	risk factor	VUS	VUS
	20	1163170	T	G	PSMF1	rs751159916	Missense variant	ENST00000335877.11:c.592T>G	ENSP00000338039.6:p.Leu198Val		8.993e-7	5/7	Deleterious (0)	Probably damaging (1)	23	het	Not reported	VUS	VUS
**03**	22	50625992	G	C	ARSA	rs1323138489	Missense variant	ENST00000216124.10:c.1051C>G	ENSP00000216124.5:p.Pro351Ala		0	6/8	Deleterious (0.01)	Probably damaging (0.99)	26.2	het	Not reported	VUS	VUS
**16**	12	40305801	G	A	LRRK2		Missense variant	ENST00000298910.12:c.3794G>A	ENSP00000298910.7:p.Gly1265Asp			28/51	Deleterious (0)	Probably damaging (1)	28.6	het	VUS	VUS	VUS
**21**	2	74367396	C	T	DCTN1	rs377183051	Missense variant	ENST00000628224.3:c.2209G>A	ENSP00000487279.2:p.Glu737Lys	0.0001029	4.397e-05	19/32	Deleterious low confidence (0)		27	het	VUS	VUS	VUS
**27**	1	20644665	A	T	PINK1	rs139226733&CM053378	Missense variant	ENST00000321556.5:c.952A>T	ENSP00000364204.3:p.Met318Leu	0.0008599	0.0007237	4/8	Deleterious (0.01)	Benign (0.073)	24.4	het	VUS	VUS	VUS
**32**	15	89325550	G	A	POLG	rs144374017&CM078774	Missense variant	ENST00000268124.11:c.1849C>T	ENSP00000268124.5:p.Arg617Cys	4.411e-05	2.639e-05	10/23	Deleterious low confidence (0)	Benign (0.13)	21.3	het	VUS	VUS	VUS

**47**	22	50627165	C	T	ARSA	rs80338815&CS910416&COSV99049447	Splice donor variant	ENST00000216124.10:c.465+1G>A		0.0007351	0.001049	2/7			29.5	het	P	P	P
**66**	22	50627165	C	T	ARSA	rs80338815&CS910416&COSV99049447	Splice donor variant	ENST00000216124.10:c.465+1G>A		0.0007351	0.001049	2/7			29.5	het	P	P	P
**67**	15	89325639	G	A	POLG	rs113994096&CM031330&COSV105090786	Missense variant	ENST00000268124.11:c.1760C>T	ENSP00000268124.5:p.Pro587Leu	0.002558	0.002698	10/23	Deleterious low confidence (0)	Probably damaging (0.998)	29.8	het	P/LP/VUS	LB	LP
	15	89318587	G	A	POLG	rs2307440&CM060433&COSV51525791	Missense variant	ENST00000268124.11:c.3436C>T	ENSP00000268124.5:p.Arg1146Cys	0.0002499	0.0002639	21/23	Deleterious low confidence (0.01)	Probably damaging (0.999)	32	het	VUS/B	VUS	VUS
**82**	1	20644570	C	T	PINK1	rs146691996	Missense variant	ENST00000321556.5:c.857C>T	ENSP00000364204.3:p.Pro286Leu	0.00002625	0.00001779	4/8	Deleterious (0.01)	Probably damaging (0.983)	27.4	het	VUS	VUS	VUS
**84**	15	89317378	T	C	POLG	COSV51521900	Missense variant	ENST00000268124.11:c.3641A>G	ENSP00000268124.5:p.Gln1214Arg			22/23	Deleterious low confidence (0.05)	Benign (0.005)	21.5	het	Not reported	VUS	VUS
**85**	20	1165052	C	T	PSMF1	rs1378117086&COSV55674659	Missense variant	ENST00000335877.11:c.788C>T	ENSP00000338039.6:p.Pro263Leu		9.892e-06	7/7	Deleterious (0)	Probably damaging (0.969)	33	het	Not reported	VUS	VUS
**91**	6	161785819	C	T	PRKN	rs769230602&COSV58202909&COSV58220892&COSV58225018	Missense variant	ENST00000366898.6:c.824G>A	ENSP00000355865.1:p.Arg275Gln	1.47e-05	3.522e-05	7/12	Deleterious (0)	Probably damaging (0.999)	28.5	het	VUS	LP	VUS
	15	61915767	G	T	VPS13C	rs201717998&COSV51314301	Missense variant	ENST00000644861.2:c.8311C>A	ENSP00000493560.2:p.Pro2771Thr	5.88e-05	7.035e-05	61/85	Deleterious low confidence (0)	Probably damaging (1)	26.3	het	Not reported	VUS	VUS

**94**	22	38132992	G	A	PLA2G6	rs1301929071&COSV59270148	Missense variant	ENST00000332509.8:c.916C>T	ENSP00000333142.3:p.Arg306Trp	1.47e-05	0	7/17	Tolerated(0.08)	Possibly damaging (0.708)	25	het	VUS	VUS	VUS
**95**	12	40363440	C	T	LRRK2	rs113511708&CM054773	Missense variant	ENST00000298910.12:c.7067C>T	ENSP00000298910.7:p.Thr2356Ile	0.0003533	0.000282	48/51	Deleterious (0.03)	Benign (0.013)	21.3	het	VUS/LB	VUS	VUS
**97**	14	54865436	T	C	GCH1		Missense variant	ENST00000491895.7:c.344A>G	ENSP00000419045.2:p.Asp115Gly			2/6	Deleterious low confidence (0.05)	Benign (0.04)	21.9	het	Not reported	VUS	VUS
**98**	22	50627245	C	G	ARSA		Missense variant	ENST00000216124.10:c.386G>C	ENSP00000216124.5:p.Gly129Ala			2/8	Deleterious (0)	Probably damaging (0.999)	24.6	het	Not reported	P	LP
**101**	3	48599673	T	C	UQCRC1		Missense variant	ENST00000203407.6:c.1340A>G	ENSP00000203407.5:p.Lys447Arg			12/13	Tolerated(0.09)	Probably damaging (0.975)	22.2	het	Not reported	VUS	VUS
**116**	13	51974355	G	A	ATP7B	rs121907999&CM960119	Stop gained	ENST00000242839.10:c.865C>T	ENSP00000242839.5:p.Gln289Ter		5.351e-05	2/21			34	het	P/LP	P	P
**119**	7	56106406	C	A	CHCHD2	rs749822788	Missense variant	ENST00000395422.4:c.8G>T	ENSP00000378812.3:p.Arg3Leu		0	1/4	Deleterious (0.04)	Unknown (0)	27.9	het	Not reported	VUS	VUS
**120**	15	89330106	T	A	POLG	rs138929605&CM083025	Missense variant	ENST00000268124.11:c.830A>T	ENSP00000268124.5:p.His277Leu	0.0007056	0.0007135	3/23	Deleterious low confidence (0.03)	Benign (0.059)	23	het	P/LP/VUS/LB	LP	LP
**127**	11	2167875	T	G	TH	rs1002471580&CM124990	Missense variant	ENST00000352909.8:c.635A>C	ENSP00000325951.4:p.Gln212Pro			5/13	Deleterious (0.05)	Possibly damaging (0.87)	25.6	het	VUS	LP	VUS
**129**	15	89330106	T	A	POLG	rs138929605&CM083025	Missense variant	ENST00000268124.11:c.830A>T	ENSP00000268124.5:p.His277Leu	0.0007056	0.0007135	3/23	Deleterious low confidence (0.03)	Benign (0.059)	23	het	P/LP/VUS/LB	LP	LP

**132**	13	51968596	C	T	ATP7B	rs192957846&COSV99070189	Missense variant	ENST00000242839.10:c.1555G>A	ENSP00000242839.5:p.Val519Met	0.0006028	0.001123	4/21	Deleterious low confidence (0)		24.6	het	VUS	VUS	VUS
**158**	18	45887809	G	A	EPG5	rs188800635&COSV104612323	Missense variant	ENST00000282041.11:c.5051C>T	ENSP00000282041.4:p.Thr1684Met	1.47e-05	9.057e-06	29/44	Deleterious (0)	Probably damaging (0.999)	26.2	het	VUS	VUS	VUS
**160**	15	61878747	C	G	VPS13C	rs759376367	Splice acceptor variant	ENST00000644861.2:c.10003-1G>C			1.834e-05	73/84			35	het	Not reported	LP	LP
	10	71818633	G	A	PSAP		Missense variant	ENST00000394936.8:c.1523C>T	ENSP00000378394.3:p.Thr508Ile			13/14	Deleterious (0)	Probably damaging (0.958)	27.5	het	Not reported	VUS	VUS
**164**	13	51944145	G	T	ATP7B	rs76151636&CM930059	Missense variant	ENST00000242839.10:c.3207C>A	ENSP00000242839.5:p.His1069Gln	0.001676	0.001281	14/21	Deleterious low confidence (0)		22.8	het	P	P	P
**180**	6	146549631	C	T	RAB32	rs775962937	Missense variant	ENST00000367495.4:c.418C>T	ENSP00000356465.3:p.Leu140Phe		0.0001319	2/3	Deleterious low confidence (0)	Probably damaging (1)	27.1	het	Not reported	VUS	VUS
**182**	1	20644665	A	T	PINK1	rs139226733&CM053378	Missense variant	ENST00000321556.5:c.952A>T	ENSP00000364204.3:p.Met318Leu	0.0008599	0.0007237	4/8	Deleterious (0.01)	Benign (0.073)	24.4	het	VUS	VUS	VUS
**183**	21	32664963	C	A	SYNJ1	rs145978776	Missense variant	ENST00000674351.1:c.2254G>T	ENSP00000501530.1:p.Asp752Tyr	0.0003234	0.0002816	18/33	Deleterious low confidence (0)	Probably damaging (0.97)	29	het	VUS	VUS	VUS
**185**	6	161785820	G	A	PRKN	rs34424986&CM991007&COSV58220231	Missense variant	ENST00000366898.6:c.823C>T	ENSP00000355865.1:p.Arg275Trp	0.00341	0.003302	7/12	Deleterious (0)	Probably damaging (1)	26.1	het	P	LP	P
**192**	15	61909119	C	T	VPS13C	rs758948442	Missense variant	ENST00000644861.2:c.8851G>A	ENSP00000493560.2:p.Gly2951Ser		1.78e-05	65/85	Deleterious low confidence (0)	Probably damaging (0.982)	25.6	het	Not reported	VUS	VUS

**205**	15	89325639	G	A	POLG	rs113994096&CM031330&COSV105090786	Missense variant	ENST00000268124.11:c.1760C>T	ENSP00000268124.5:p.Pro587Leu	0.002558	0.002698	10/23	Deleterious low confidence (0)	Probably damaging (0.998)	29.8	het	P/LP/VUS	LB	LP
**219**	13	51968596	C	T	ATP7B	rs192957846&COSV99070189	Missense variant	ENST00000242839.10:c.1555G>A	ENSP00000242839.5:p.Val519Met	0.0006028	0.001123	4/21	Deleterious low confidence (0)		24.6	het	VUS	VUS	VUS
**226**	1	16992353	G	A	ATP13A2	rs376070950&COSV58703214&COSV99045022	Missense variant	ENST00000326735.13:c.1895C>T	ENSP00000327214.8:p.Ser632Leu	4.409e-05	3.616e-05	18/29	Deleterious (0)	Probably damaging (1)	35	het	VUS	VUS	VUS
**227**	15	61921963	C	G	VPS13C		Missense variant	ENST00000644861.2:c.7046G>C	ENSP00000493560.2:p.Trp2349Ser			55/85	Deleterious low confidence (0)	Benign (0.117)	24.2	het	Not reported	VUS	VUS
**231**	22	32491131	G	A	FBXO7	rs771599282	Missense variant	ENST00000266087.7:c.917G>A	ENSP00000266087.7:p.Arg306His	2.000e-04	8.069e-05	6/9	Deleterious (0)	Probably damaging (0.984)	31	het	Not reported	VUS	VUS
**240**	22	38140045	C	T	PLA2G6	rs776469475	Missense variant	ENST00000332509.3:c.734G>A	ENSP00000333142.3:p.Arg245Gln	0	1.80e-05	5/17	Tolerated(0.11)	Possibly damaging (0.838)	23.7	het	Not reported	VUS	VUS
**253**	13	51946372	G	A	ATP7B	rs41292782&CM053119	Missense variant	ENST00000242839.10:c.2972C>T	ENSP00000242839.5:p.Thr991Met	0.00291	0.002359	13/21	Deleterious low confidence (0)		27.7	het	P/LP/VUS	P	VUS
**255**	15	61991096	T	C	VPS13C	rs143639809	Splice acceptor variant	ENST00000261517.5:c.1484-2A>G		0.0019385	0.0015954	17/84			34	het	B/LB	LP	LB
	12	40335031	G	A	LRRK2	rs77428810&CM054774&COSM1322529&COSM1322530	Missense variant	ENST00000298910.7:c.5822G>A	ENSP00000298910.7:p.Arg1941His	6.668e-05	0.00020597	40/51	Tolerated(0.07)	Benign (0.055)	23	het	VUS	VUS	VUS

**259**	22	50626231	C	T	ARSA	rs573456864&COSV53351533	Missense variant	ENST00000216124.10:c.902G>A	ENSP00000216124.5:p.Arg301Gln	1.470e-05	1.439e-05	5/8	Deleterious (0)	Probably damaging (0.939)	32	het	VUS	VUS	LP
**261**	6	161785877	G	A	PRKN	rs150562946&CM991006	Missense variant	ENST00000366898.1:c.766C>T	ENSP00000355865.1:p.Arg256Cys	0.00079957	0.0006274	7/12	Deleterious (0.01)	Probably damaging (0.964)	32	het	VUS	LP	VUS
**272**	1	20644665	A	T	PINK1	rs139226733&CM053378	Missense variant	ENST00000321556.5:c.952A>T	ENSP00000364204.3:p.Met318Leu	0.0008599	0.0007237	4/8	Deleterious (0.01)	Benign (0.073)	24.4	het	VUS	VUS	VUS
**276**	6	161350211	C	T	PRKN	rs760223151&CM096653&COSM6922828	Missense variant	ENST00000366898.1:c.1286G>A	ENSP00000355865.1:p.Gly429Glu		3.628e-05	12/12	Deleterious (0)	Probably damaging (0.992)	33	het	Not reported	LP	VUS
	13	51946372	G	A	ATP7B	rs41292782&CM053119	Missense variant	ENST00000242839.10:c.2972C>T	ENSP00000242839.5:p.Thr991Met	0.00291	0.002359	13/21	Deleterious low confidence (0)		27.7	het	P/LP/VUS	P	VUS
**281**	15	61911970	A	T	VPS13C	rs199917836	Missense variant	ENST00000261517.5:c.8585T>A	ENSP00000261517.5:p.Leu2862His	0.00013329	0.00012614	63/85	Deleterious (0)	Probably damaging (0.999)	28	het	Not reported	VUS	VUS
**285**	15	89325639	G	A	POLG	rs113994096&CM031330&COSV105090786	Missense variant	ENST00000268124.11:c.1760C>T	ENSP00000268124.5:p.Pro587Leu	0.002558	0.002698	10/23	Deleterious low confidence (0)	Probably damaging (0.998)	29.8	het	P/LP/VUS	LB	LP

ACMG: American College of Medical Genetics and Genomics Alt: Alternate B: Benign CADD: Combined Annotation Dependent Depletion Chr: Chromosome het: heterozygous HGVS: Human Genome Variation Society LB: Likely Benign LP: Likely Pathogenic NFE: Non-Finnish Europeans P: Pathogenic Ref: Reference VUS: Variant of Uncertain Significance

Supplemental Table S3: Contingency tables for the association tests reported in Table 3

CHCHD2 c.248_249insG p.(Phe84LeufsTer6)	PD	Controls
Mutants	2	2
Non-mutants	978	25,682

ARSA c.386G>C p.(Gly129Ala)	PD	Controls
Mutants	1	0
Non-mutants	979	25,684

ARSA c.465 + 1 G>A	PD	Controls
Mutants	5	81
Non-mutants	975	25,603

ARSA c.465 + 1 G>A (MDCS only)	PD	Controls
Mutants	3	81
Non-mutants	977	25,603

ARSA c.902G>A p.(Arg301Gln)	PD	Controls
Mutants	1	13
Non-mutants	979	25,671

ARSA c.1051C>G p.(Pro351Ala)	PD	Controls
Mutants	1	6
Non-mutants	979	25,678

Supplemental Figure S1: Workflow diagram of the bioinformatic analyses

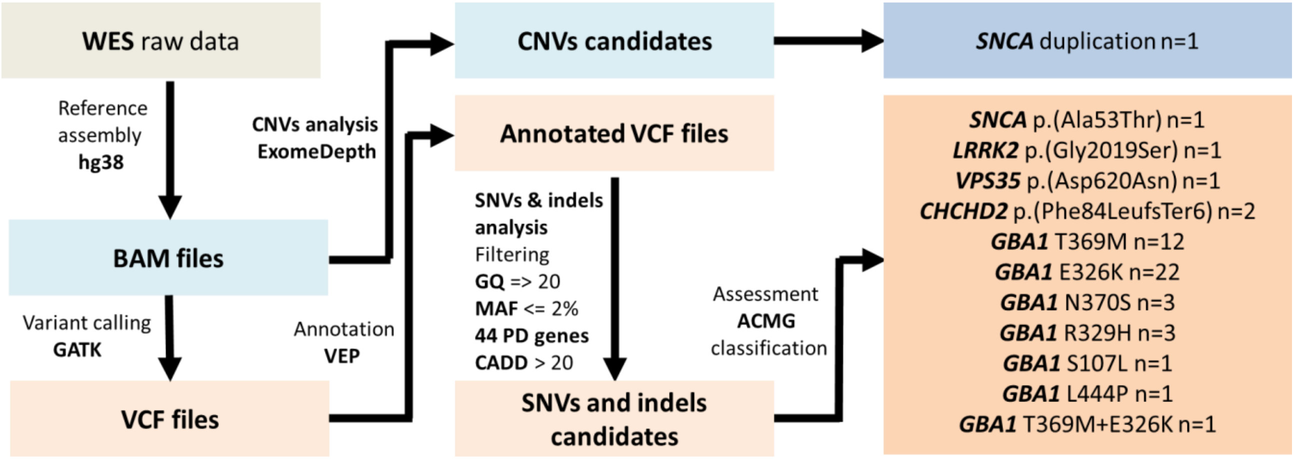

Supplemental Figure S2: 123I-ioflupane SPECT of patient P193 with VPS35 p.(Asp620Asn) at age 51

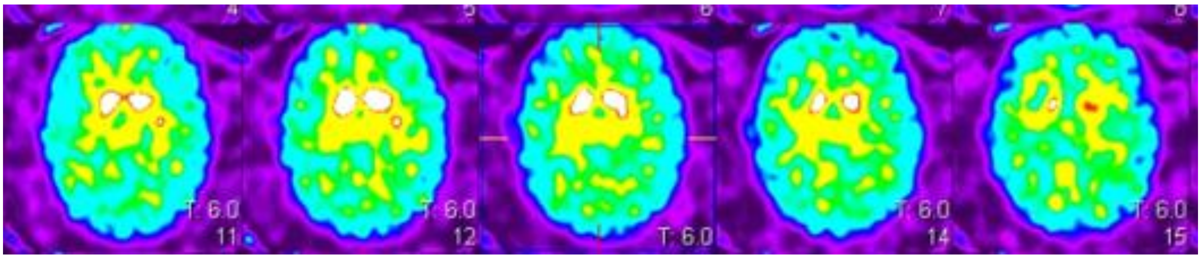

Supplemental Figure S3: Graphs of SNV array assay of *SNCA* duplications identified in one member of the Lister family (A) and in the PD patient of this study (B).

A

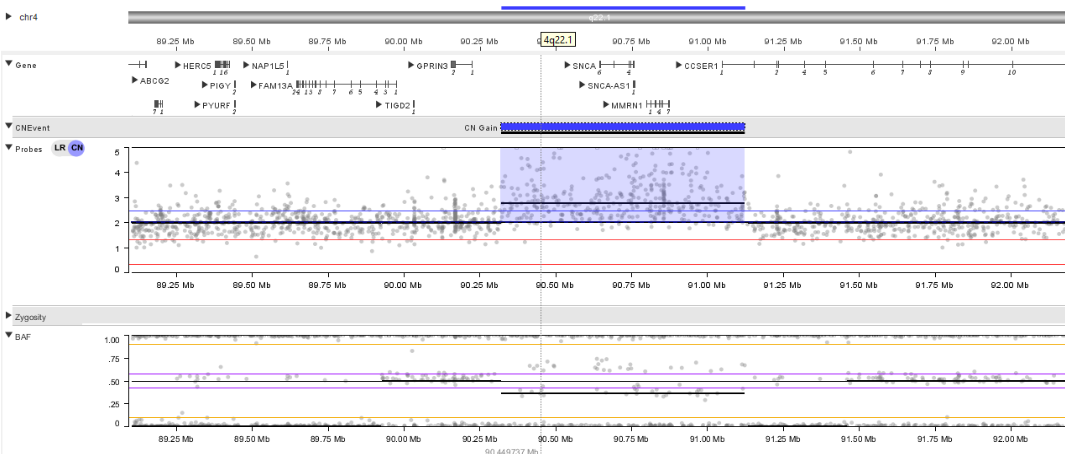

B

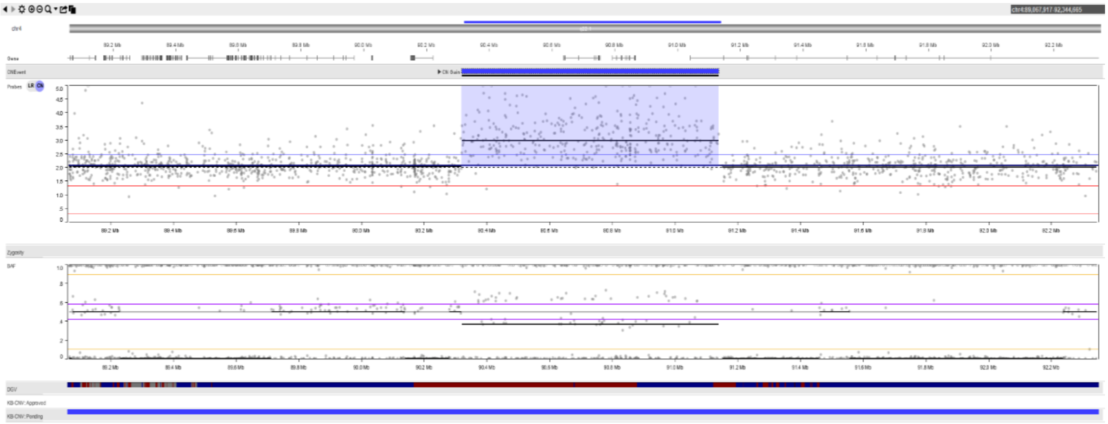

References to supplement:

1. Stefanou, M. I.; Katsaros, V. K.; Pepe, G.; Theodorou, A.; Stefanou, D.; Koropouli, E.; Paraskevas, G. P.; Tsivgoulis, G. Early-Onset Parkinson's Disease in a Patient With a De Novo Frameshift Variant of the ANKRD11 Gene and KBG Syndrome. *J Clin Neurol* **2025**, *21*, 153-155.

2. Lee, J. S.; Kanai, K.; Suzuki, M.; Kim, W. S.; Yoo, H. S.; Fu, Y.; Kim, D. K.; Jung, B. C.; Choi, M.; Oh, K. W.; et al. Arylsulfatase A, a genetic modifier of Parkinson's disease, is an alpha-synuclein chaperone. *Brain* **2019**, *142*, 2845-2859.

3. Mencacci, N. E.; Minakaki, G.; Maroofian, R.; De Pace, R.; Paimboeuf, A.; Shannon, P.; Chitayat, D.; Magrinelli, F.; Peng, W. J.; Chatterjee, D.; et al. Pathogenic variants in BORCS5 Cause a Spectrum of Neurodevelopmental and Neurodegenerative Disorders with Lysosomal Dysfunction. *medRxiv* **2025**.

4. Liu, Z.; Yang, N.; Dong, J.; Tian, W.; Chang, L.; Ma, J.; Guo, J.; Tan, J.; Dong, A.; He, K.; et al. Deficiency in endocannabinoid synthase DAGLB contributes to early onset Parkinsonism and murine nigral dopaminergic neuron dysfunction. *Nat Commun* **2022**, *13*, 3490.

5. Sun, Q. Y.; Tang, F. L.; Zhou, Y.; Pan, H. X.; Zhou, X.; Zhao, Y. W.; He, R. C.; Zeng, S.; Wang, J. P.; Lin, W.; et al. Biallelic Variants in EPG5 Gene Are Associated with Parkinson's Disease. *Ann Neurol* **2025**.

6. Skuladottir, A. T.; Tragante, V.; Sveinbjornsson, G.; Helgason, H.; Sturluson, A.; Bjornsdottir, A.; Jonsson, P.; Palmadottir, V.; Sveinsson, O. A.; Jensson, B. O.; et al. Loss-of-function variants in ITSN1 confer high risk of Parkinson's disease. *NPJ Parkinsons Dis* **2024**, *10*, 140.

7. Magrinelli, F.; Tesson, C.; Angelova, P. R.; Salazar-Villacorta, A.; Rodriguez, J. A.; Scardamaglia, A.; Chung, B. H.; Jaconelli, M.; Vona, B.; Esteras, N.; et al. PSMF1 variants cause a phenotypic spectrum from early-onset Parkinson's disease to perinatal lethality by disrupting mitochondrial pathways. *medRxiv* **2024**.

8. Kuipers, D. J. S.; Carr, J.; Bardien, S.; Thomas, P.; Sebate, B.; Breedveld, G. J.; van Minkelen, R.; Brouwer, R. W. W.; van Ijcken, W. F. J.; van Slegtenhorst, M. A.; et al. PTRHD1 Loss-of-function mutation in an african family with juvenile-onset Parkinsonism and intellectual disability. *Mov Disord* **2018**, *33*, 1814-1819.

9. Gustavsson, E. K.; Follett, J.; Trinh, J.; Barodia, S. K.; Real, R.; Liu, Z.; Grant-Peters, M.; Fox, J. D.; Appel-Cresswell, S.; Stoessl, A. J.; et al. RAB32 Ser71Arg in autosomal dominant Parkinson's disease: linkage, association, and functional analyses. *Lancet Neurol* **2024**, *23*, 603-614.

10. Percetti, M.; Franco, G.; Monfrini, E.; Caporali, L.; Minardi, R.; La Morgia, C.; Valentino, M. L.; Liguori, R.; Palmieri, I.; Ottaviani, D.; et al. TWNK in Parkinson's Disease: A Movement Disorder and Mitochondrial Disease Center Perspective Study. *Mov Disord* **2022**, *37*, 1938-1943.
